# Impact of Coronavirus Disease (COVID-19) Pandemic on Psychological Well-Being of the Pakistani General Population

**DOI:** 10.3389/fpsyt.2020.564364

**Published:** 2021-01-12

**Authors:** Adeel Ahmed Khan, Fahad Saqib Lodhi, Unaib Rabbani, Zeeshan Ahmed, Saidul Abrar, Saamia Arshad, Saadia Irum, Muhammad Imran Khan

**Affiliations:** ^1^Saudi Board Preventive Medicine, Ministry of Health, Mecca, Saudi Arabia; ^2^Department of Community Medicine, Women Medical and Dental College Abbottabad, Abbottabad, Pakistan; ^3^Department of Epidemiology and Biostatistics, School of Public Health, Tehran University of Medical Sciences, Tehran, Iran; ^4^Family Medicine Academy, Qassim Health Cluster, Buraidah, Saudi Arabia; ^5^Madina Sector, Preventive Medicine Department, Ministry of Health, Bisha, Saudi Arabia; ^6^Department of Community Medicine, Gajju Khan Medical College Swabi, Swabi, Pakistan; ^7^Ayub Medical College, Abbottabad, Pakistan; ^8^Department of Gynecology and Obstetrics, Ayub Medical College, Abbottabad, Pakistan; ^9^Ripha Institute of Pharmaceutical Sciences, Ripha International University, Islamabad, Pakistan

**Keywords:** COVID-19, mental health, psychological impact, well-being, Pakistan

## Abstract

**Background and Objectives:** In order to curb the spread of coronavirus disease 2019 (COVID-19), the countries took preventive measures such as lockdown and restrictions of movements. This can lead to effects on mental health of the population. We studied the impact of COVID-19 on psychological well-being and associated factors among the Pakistani general population.

**Methods:** An online cross-sectional survey was conducted between 26th April and 15th May and included participants from all over the Pakistan. Attitudes and worriedness about COVID-19 pandemic were assessed using a structured questionnaire. A validated English and Urdu version of the World Health Organization Well-Being Index (WHO-5) was used to assess the well-being. Factor analysis was done to extract the attitude item domains. Logistic regression was used to assess the factors associated with poor well-being.

**Results:** A total of 1,756 people participated in the survey. Almost half 50% of the participants were male, and a similar proportion was employed. About 41% of the participants were dependent on financial sources other than salary. News was considered a source of fear as 72% assumed that avoiding such news may reduce the fear. About 68% of the population was worried about contracting the disease. The most common coping strategies used during lockdown were spending quality time with family, eating healthy food, adequate sleep, and talking to friends on phone. Prevalence of poor well-being was found to be 41.2%. Female gender, being unemployed, living in Sindh and Islamabad Capital Territory (ICT), fear of COVID-19, and having chronic illness were significantly associated with poor well-being. Similarly, coping strategies during lockdown (doing exercise; spending time with family; eating healthy food; having good sleep; contributing in social welfare work and spending time on hobbies) were also significantly associated with mental well-being.

**Conclusion:** We found a high prevalence 41.2% of poor well-being among the Pakistani general population. We also investigated risk factors of poor well-being which included female gender, unemployment, being resident of ICT and Sindh, fear, chronic illness, and absence of coping strategies. This calls for immediate action at population level in the form of targeted mass psychological support programs to improve the mental health of population during the COVID-19 crises.

## Introduction

The Corona virus disease 2019 (COVID-19) emerged in Wuhan, Hubei province of China, where a large number of patients presented with pneumonia of unknown etiology (1). Later the disease spread nationwide and across the world between December 2019 to early 2020 (2). The World Health Organization (WHO) announced the outbreak of the novel corona virus disease as a public health emergency of international concern under the International Health Regulations (IHR) on January 30, 2020, and the disease was declared a pandemic on March 11, 2020, affecting 169 countries and almost all continents (3).

COVID-19 not only posed serious threats to physical health but also triggered negative impacts on the social, psychological, and mental health of the population (4). Psychological and mental health refers to the state of people in which they realize their own ability to cope with life stressors (5). Many factors affect the psychological and mental health of the population, for instance uncertainty of the illness, social distancing, self-isolation, and quarantine (6). A nationwide survey conducted in China on 31 January 2020 revealed that the mean score of the COVID-19 Peritraumatic Distress Index (CPDI) was 23.65 (±15.45) which inquired about the frequency of anxiety, depression, phobias, cognitive change, avoidance and compulsive behavior, and loss of social functioning in the population. Almost 35% of the respondents experienced psychological and mental health problems (7). Another survey conducted in China analyzed the psychological impact of COVID 19 among the elderly population which revealed that seniors of all age segments have depression and anxiety issues (8).

Pakistan has been in the state of high alert since February 2020 when the first case was notified in the country. Government and health professionals advised for preventive measures to prevent the spread of the disease (6). These measures were later intensified with the increasing number of cases and local transmission. The government implemented complete lockdown, closure of businesses and mosques, restriction of movements, and working at home to promote social distancing and curb the spread of disease. These precautionary measures such as social distancing, staying at home, and lockdown may lead to psychological and mental health problems (6). A study conducted in Karachi, Pakistan, in March 2020 highlighted psychological problems such as increase in anxiety level and fear and changes in the behavior to ensure safety (9).

Studies have confirmed that the outbreak of COVID-19 is associated with various psychological problems, and these may continue even after outbreak is over. It is therefore important to estimate the burden of psychological problems and identify the high-risk groups in the population who may need psychological support during this crisis.

This study was a nationwide survey aimed to analyze the psychological impact of the COVID-19 epidemic in the general population of Pakistan during the outbreak. Findings of this study will be helpful in targeting the vulnerable population having psychological problems and developing a better, scientifically sound and nationwide strategic plan for comprehensive psychological crisis management.

## Methods

We conducted an online survey among the general population of Pakistan. According to the Population Census of 2017, the total population of Pakistan is 207 million with a growth rate of 2.4%. The administrative units of Pakistan consist of four provinces, i.e., Punjab, Sindh, Baluchistan, and Khyber Pakhtunkhwa, along with two autonomous territories Azad Kashmir and Gilgit-Baltistan and one federal territory, Islamabad (10).

Due to the strict lockdown all over the country to implement social distancing and to control the disease spread, it was not possible to conduct one-to-one interviews in the community. Therefore, we decided to conduct an online survey by using all possible means of contacting the general population. Convenience and snowball sampling strategy was used to enroll the general population of Pakistan. Participants were approached through a web-based self-administered questionnaire which was formulated on Google forms. We circulated the survey link to general population through WhatsApp, Facebook, and email addresses between 26th April and 15th May 2020.

### Sample Size Calculation

Sample size was calculated using open epi sample size calculator (11). We assumed a proportion of 50% of the population to have poor well-being. This 50% proportion would provide maximum variance and sample size. At 95% confidence level and 4% absolute precision, the sample size calculated was 600 participants. We used a design effect of 2.5 to inflate our sample to capture population variability which increased to a sample of 1,500. We further inflated our sample by 20% to account for incomplete and missing data so the final sample size required was 1,800 participants.

### Data Collection Instruments, Measures, and Variables

Data was collected using a structured questionnaire in English and local language Urdu. The questionnaire was divided into three sections and had a total of 27 questions. The first two questions before the start of demographic information were about their willingness to participate and language selection. The socio-demographic information collected in section 1 included gender, age, city, education, marital status, employment, employment type, family type, and financial support.

The attitudes of the Pakistani general population related to COVID-19 were collected in section 2 and were assessed regarding the following aspects: believing in successful control against COVID-19, believing that Pakistan can win the battle against COVID-19, believing that stopping oneself from watching news will help in decreasing the fear, and believing that the unauthentic and unverified information spreading through forwarded messages is increasing panic about COVID-19. A five-point Likert scale (strongly disagree to strongly agree) was used for all these questions.

To assess the worriedness regarding COVID-19, we asked five questions in section 3 regarding the following aspects: worried about eventually contracting COVID-19 despite taking preventive measures; worried about not being able to survive if they get infected with COVID-19; worried that if they contract the infection, drugs/treatment will have no effect; worried that they will pass the virus onto their family; and not worried of contracting it because they are already old and have lived their life the best way they could. The five-point Likert scale of worriedness was used, which included “no worry at all,” “mildly worried,” “somewhat worried,” “moderately worried,” and “extremely worried.”

### WHO-5 Well-Being Index

The five-item World Health Organization Well-Being Index (WHO-5) is among the most commonly used questionnaires assessing subjective psychological well-being (12). It is a short form of the WHO-10 item and 28-item rating scales (12, 13). WHO-5 can be used as a screening tool for depressive symptoms, monitoring emotional well-being and psychological well-being (14, 15). The WHO-5 items were the following: I have felt cheerful and in good spirits, I have felt calm and relaxed, I have felt active and vigorous, I woke up feeling fresh and rested, and my daily life has been filled with things that interest me. The response alternatives were “all of the time = 5,” “most of the time = 4,” “more than half of the time = 3,” “less than half of the time = 2,” “some of the time = 1,” or “at no time = 0.” The respondents were asked to rate how well each of the five statements applies to them when considering the last 14 days. Each of the five items is scored from 5 (all of the time) to 0 (none of the time). The raw score therefore theoretically ranges from 0 (absence of well-being) to 25 (maximal well-being). The raw score ranging from 0 to 25 was multiplied by four to give the final score from 0 representing the worst imaginable possible well-being to 100 representing the best imaginable well-being. We used official Urdu version of WHO-5 well-being scale (16).

### Statistical Analysis

Data was downloaded as Microsoft Excel sheet and then imported to IBM SPSS for Windows, v. 22.0 (IBM Corp., Armonk, USA), for analysis. Mean and standard deviations were calculated for quantitative variables such as age, attitude domains and well-being scores. Categorical variables such as gender, marital status, education, employment status, type of employment, family type, region, financial support during lockdown, and disease status were expressed as frequencies and percentages.

The proportion for the attitudes, worriedness, and coping strategies used by the participants at the time of lockdown was determined by using frequencies of individual questions. The five-point Likert scale was converted into three-point responses such as the following: “strongly disagree” and “disagree” were merged as “disagree;” “strongly agree” and “agree” were merged as “agree;” and a middle category was “don't know.” Similarly, the five-point Likert scale of worriedness was also converted into three-point responses; that is, “No worry at all” and “Mildly worried” and “Somewhat worried” were merged as “Somewhat worried,” and a last category was “Extremely worried.” Factor analysis was done to explore the attitude questions and give them a factor solution. Construct validity for attitude domain items was analyzed using exploratory factor analysis (EFA). To find the best fit to the data, orthogonal (varimax) rotation was used in our factor analysis. Factor loadings of more than 0.40 were considered satisfactory.

Prevalence of poor well-being was determined using a cutoff of ≤50 score on the 100-point WHO-5 well-being scale (17). Univariate and multivariate logistic regression models were developed to explore the factors associated with poor well-being among general population. Variables were included in the multivariate model based on contribution in the overall model assessed by −2 log likelihood ratios. Crude and adjusted odds ratio (aOR) along with associated 95% confidence intervals (CI) were calculated.

### Ethical Approval

The ethical approval of the study was sought from the Ethics Review Committee of Women Medical College, Abbottabad (20204-2 CMD-ERC-20). The first page of the online form described the purpose of the study and consent was taken on that page from all the participants.

## Results

A total of 1,756 individuals participated in the survey. The mean age of the participants was 31.5 ± 10.8 years, and half (49.9%) were male. More than half (58%) had post-graduation or a professional degree. About 50% were employed, 32% were students, and 6.2% were unemployed. Among the employed, majority (48%) had a private job. The highest proportion of participants was from Khyber Pakhtunkhwa (KPK) 37% followed by Sindh 32%. The most common financial support during lockdown was salary (59%), followed by savings (15.4%). The most common chronic disease was hypertension (10.9%) while prevalence of any chronic illness was 23.8% (Table 1).

**Table 1:** Socio-demographic characteristics of the study participants (*n* = 1,756).

**Characteristic**	***n* (%)**
**Age (*****n*** **= 1,728)**	
Mean (SD)	31.5 (10.8)
**Gender (*****n*** **= 1,730)**	
Male	863 (49.9)
Female	876 (50.1)
**Marital status (*****n*** **= 1,745)**	
Single	828 (47.4)
Ever married	917 (52.6)
**Education (*****n*** **= 1,733)**	
Postgraduate	1,010 (58.3)
Graduate	405 (23.4)
Intermediate	259 (14.9)
Other	59 (3.4)
**Employment status (*****n*** **= 1,746)**	
Employed	880 (50.4)
Student	556 (31.8)
Housewife	178 (10.2)
Retired	23 (1.3)
Unemployed	109 (6.2)
**Type of employment (*****n*** **= 871)**	
Private job	419 (48.1)
Government job	340 (39.0)
Business	73 (8.4)
Small business/vendor	21 (2.4)
Part time work	10 (1.1)
Laborer	8 (0.9)
**Family type (*****n*** **= 1,745)**	
Joint	905 (51.9)
Nuclear	840 (48.1)
**Region (*****n*** **= 1,728)**	
Punjab	287 (16.6)
Sindh	553 (32.0)
Khyber Pakhtunkhwa	641 (37.1)
Baluchistan	53 (3.1)
Islamabad Capital Territory	136 (7.9)
Other	58 (3.7)
**Financial support during lockdown (*****n*** **= 1,724)**	
Salary	1,013 (58.8)
Savings	266 (15.4)
Business earning	218 (12.6)
Support from friends and family	120 (7.0)
No source	62 (3.6)
Loan	29 (1.7)
Pension	16 (0.9)
**Disease status**	
Any chronic illness	418 (23.8)
Cardiac disease	38 (2.2)
Hypertension	191 (10.9)
Diabetes	88 (5.0)
Others	206 (11.7)

Table 2 presents the attitude and worries related to COVID-19 among the Pakistani population. About 69% and 73% participants believed that COVID-19 will be controlled globally and in Pakistan, respectively. Seventy-two percent assumed that avoiding news may help reduce the fears of COVID-19. A high proportion (90%) believed that unauthentic information through social media is adding to the panic about COVID-19. About two-thirds (68%) of the population was somewhat worried about contracting the disease even with preventive measures. Six percent were worried about surviving after the infection and no effects of treatment. A little less than half (43%) were extremely worried about transmitting infection to family members.

**Table 2:** Attitudes and worriedness of Pakistani general population related to COVID-19 (*n* = 1,756).

**Variable**	**n (%)**
**ATTITUDES**	
**COVID-19 will be controlled (*****n*** **= 1,744)**	
Agree	1,207 (69.2)
Don't know	328 (18.8)
Disagree	209 (12.0)
**Pakistan will be able to control COVID-19 (*****n*** **= 1,747)**	
Agree	1,270 (72.7)
Don't know	302 (17.3)
Disagree	175 (10.0)
**Stopping yourself from watching news will help in decreasing the fear (*****n*** **= 1,746)**	
Agree	1,259 (72.1)
Don't know	105 (6.0)
Disagree	382 (21.9)
**Unauthentic information through social media is increasing panic about COVID-19 (*****n*** **= 1,741)**	
Agree	1,577 (90.6)
Don't know	63 (3.6)
Disagree	101 (5.8)
**Social distancing will become the new norm and we will not be able to meet people in future like we did before (*****n*** **= 1,743)**	
Agree	540 (31.0)
Don't know	272 (15.6)
Disagree	931 (53.4)
**WORRIES**	
**I will eventually contract COVID-19 despite preventive measures (*****n*** **= 1,733)**	
Not worried at all	483 (27.9)
Somewhat worried	1182 (68.2)
Extremely worried	68 (3.9)
**I will not be able to survive COVID-19 if I contract the infection (*****n*** **= 1,731)**	
Not worried at all	636 (36.7)
Somewhat worried	992 (57.3
Extremely worried	103 (6.0)
**Drugs/treatment will have no effect on me (*****n*** **= 1,716)**	
Not worried at all	709 (41.3)
Somewhat worried	903 (52.6)
Extremely worried	104 (6.1)
**I am ______ if I get the infection, I will pass onto my family (*****n*** **= 1,731)**	
Not worried at all	172 (9.9)
Somewhat worried	817 (47.2)
Extremely worried	742 (42.9)
**I am ______ about contracting it because I am old and have lived my life the best way I could (*****n*** **= 1,660)**	
Not worried at all	706 (42.5)
Somewhat worried	750 (45.2)
Extremely worried	204 (12.3)

Prior to factor extraction for attitude domain items, sampling adequacy was checked to ensure suitability for factor analysis. The Kaiser–Meyer–Olkin (KMO) test and the Bartlett test of sphericity (BTS) showed adequate sampling. The number of factors was assessed using eigenvalues >1 and a scree plot which suggested a three-factor solution and explained 57.41% of the total variance. We named the factors as controllability, misinformation, and fear related to the attitude domains of COVID-19. One item was eliminated because it did not contribute to a simple factor structure and failed to meet a minimum criterion of having a primary factor loading of 0.4 or above (Table 3). Descriptive statistics of these domains were run. Mean score was computed as the following: for the controllability domain (7.65 ± 1.80), for the misinformation domain (8.07 ± 1.69), and for the fear domain (16.03 ± 4.61), respectively, with higher scores suggesting higher degree of believability related to items of specific domains.

**Table 3:** Exploratory factor analysis of attitude domains items with Varimax rotation matrix (*n* = 1,756).

**Attitude domains items**	**F1**	**F2**	**F3**
Controllability			
COVID-19 will be controlled	*-0.071*	**0.887**	*0.069*
Pakistan will be able to control the COVID-19	*-0.128*	**0.869**	*0.081*
Misinformation			
Stopping yourself from watching news will help in decreasing the fear	*-0.042*	*0.047*	**0.806**
Unauthentic information through social media is increasing panic about COVID-19	*0.009*	*0.174*	**0.744**
Fear			
I will eventually contract COVID-19 despite preventive measures	**0.712**	*-0.144*	*0.051*
I will not be able to survive by COVID-19, if I contract the infection	**0.812**	*-0.107*	*0.004*
Drugs/treatment will have no effect on me	**0.796**	*-0.112*	*-0.034*
I am afraid if I get the infection, I will pass onto my family	**0.642**	*-0.073*	*-0.008*
I am not afraid of contracting it because I am old and have lived my life the best way I could	**0.663**	*0.021*	*0.085*
Social distancing will become the new norm and we will not be able to meet people in future like we did before^*^	*-0.174*	*0.262*	*-0.342*
% of variance: 57.41%	**27.06%**	**16.94%**	**13.41%**

*Italic indicates loading factor below 0.30. Bold indicates significant factor loading at level above 0.50*.

* *Excluded because of low loading score*.

The most common coping strategies to counter the effects of lockdown and home confinement used by the participants included spending quality time with family (83%), eating healthy food (79%), adequate sleep (77%), and talking to friends on the phone (73%). Other strategies included watching TV/movies, seeking spiritual support, working on hobbies, and reading (Table 4).

**Table 4:** Coping strategies used by participants (*n* = 1,756).

**Strategy**	***n* (%)**
Spending quality time with my family	1,464 (83.4)
Eating healthy food	1,386 (78.9)
Adequate sleep	1,348 (76.8)
Talking to friends on phone	1,293 (73.6)
Watching TV/movies	1,155 (65.8)
Seeking spiritual support	1,140 (64.9)
Working on hobbies	931 (53.0)
Reading	894 (50.9)
Contributing in social welfare work	741 (42.2)
Relaxing by meditation/yoga/exercise	725 (41.3)
Playing video games	663 (37.8)

The mean score of well-being as measured by the WHO-5 well-being scale was 55.0 ± 24.6 out of a total of 100 points. The prevalence of poor well-being was 41.2%. Detailed results of individual items of WHO-5 are presented in Supplementary Table 1.

We found that being female, being a student, being a housewife, being unemployed, having a nuclear family, living in Sindh and Islamabad Capital Territory (ICT), and having a chronic illness were significantly associated with poor well-being in the univariate analysis. Regarding the attitude domains, the controllability and fear domains were also significant in the univariate analysis. Coping strategies were also analyzed for having an association with poor well-being among the population. In the univariate analysis, individuals who were exercising; spending time with the family; reading; taking healthy food; having a good sleep; participating in social welfare work; spending time on their hobbies; seeking spiritual support; and taking to family & friends on the phone were significantly associated with better mental well-being. When adjusted for confounding effects of other variables in the multivariate model, we found that females had about 35% higher risk of poor well-being compared to males, aOR 1.35 (95% CI: 1.04–1.75). The unemployed were at about 60% higher risk of having a poor well-being compared to employed individuals, aOR 1.62 (95% CI: 1.02–2.59). Those living in the Sindh province and ICT were also at a higher risk of having a poor well-being compared to Punjab province, aOR 1.54 (95% CI: 1.10–2.16) and 1.82 (95% CI: 1.13–2.93), respectively. Similarly, individuals with any chronic illness were also at a higher risk of poor well-being, aOR 1.71 (95% CI: 1.31–2.23). Similarly, those who believed in controllability were less likely to have mental well-being, aOR 0.88 (95% CI: 0.83–0.94). Those who had fear were more likely to have poor well-being, aOR 1.06 (95% CI: 1.03–1.08). In the multivariate analysis, we also found that persons who were exercising; spending time with the family; taking healthy food; having a good sleep; participating in social welfare work; and spending time on their hobbies were significantly associated with better mental well-being. Other factors such as age, marital status, education status, and family type did not show any significant association with well-being (Table 5).

**Table 5:** Factors associated with poor well-being among general Pakistani population during COVID-19 pandemic (*n* = 1,756).

**Characteristic**	**Univariate analysis**		**Multivariate analysis**	
	**Crude OR (95% CI)**	***p*-value**	**Adjusted^**α**^ OR (95% CI)**	***p*-value**
Age^©^	0.99 (0.98–1.00)	0.113	0.99 (0.98–1.01)	0.801
**Gender**				
Male	1		1	
Female	1.46 (1.20–1.77)	**<0.001**	1.35 (1.04–1.75)	**0.025**
**Marital status**				
Single	1			
Ever married	0.88 (0.73–1.07)	0.212	–	–
**Education status**				
Postgraduate	1		1	
Graduate	1.22 (0.96–1.54)	0.100	1.22 (0.92–1.61)	0.164
Intermediate	1.15 (0.87–1.53)	0.323	1.10 (0.78–1.55)	0.595
Matric and lower	1.45 (0.85–2.46)	0.173	1.04 (0.54–2.00)	0.910
**Employment status**				
Employed	1		1	
Student	1.26 (1.01–1.57)	**0.038**	1.17 (0.81–1.68)	0.398
Housewife	1.40 (1.01–1.94)	**0.044**	0.86 (0.56–1.32)	0.491
Retired	0.68 (0.28–1.67)	0.397	0.49 (0.16–1.47)	0.204
Unemployed	1.87 (1.25–2.81)	**0.002**	1.62 (1.02–2.59)	**0.043**
**Family type**				
Joint	1		1	
Nuclear	1.27 (1.04–1.53)	**0.016**	1.12 (0.89–1.41)	0.321
**Region**				
Punjab	1		1	
Sindh	1.38 (1.03–1.86)	**0.032**	1.54 (1.10–2.16)	**0.012**
Khyber Pakhtunkhwa	1.30 (0.97–1.73)	0.076	1.18 (0.85–1.65)	0.321
Baluchistan	0.93 (0.50–1.74)	0.829	1.15 (0.55–2.37)	0.712
Islamabad Capital Territory (ICT)	1.79 (1.18–2.72)	**0.006**	1.82 (1.13–2.93)	**0.014**
Other	1.19 (0.66–2.14)	0.554	1.34 (0.71 - 2.56)	0.365
**Any chronic illness**				
No	1		1	
Yes	1.63 (1.30–2.04)	**<0.001**	1.71 (1.31–2.23)	**<0.001**
**Attitude scale domains**				
Controllability^©^	0.82 (0.78–0.87)	**<0.001**	0.88 (0.83–0.94)	**<0.001**
Misinformation^©^	0.96 (0.90–1.01)	0.135	–	–
Fear^©^	1.09 (1.06–1.11)	**<0.001**	1.06 (1.03–1.08)	**<0.001**
**COPING STRATEGY DURING LOCKDOWN**				
**Exercise**				
Yes	1		1	
No	1.99 (1.63–2.43)	**<0.001**	1.30 (1.03–1.64)	**0.028**
**Spending time with family**				
Yes	1		1	
No	2.27 (1.73–2.97)	**<0.001**	1.40 (1.00–1.95)	**0.049**
**Reading**				
Yes	1		1	
No	1.64 (1.35–1.99)	**<0.001**	1.23 (0.97–1.54)	0.081
**Eating healthy food**				
Yes	1		1	
No	2.76 (2.16–3.53)	**<0.001**	1.70 (1.25–2.31)	**0.001**
**Having good sleep**				
Yes	1		1	
No	2.19 (1.73–2.76)	**<0.001**	1.39 (1.04–1.85)	**0.026**
**Contributing in social welfare work**				
Yes	1		1	
No	1.93 (1.58–2.36)	**<0.001**	1.34 (1.06–1.71)	**0.014**
**Spending time on hobbies**				
Yes	1		1	
No	1.91 (1.58–2.33)	**<0.001**	1.47 (1.16–1.88)	**0.002**
**Watching movies**				
Yes	1		1	
No	1.00 (0.82–1.23)	0.992	0.82 (0.64–1.05)	0.119
**Playing video game**				
Yes	1			
No	0.99 (0.81–1.20)	0.901	–	–
**Seeking spiritual support**				
Yes	1			
No	1.66 (1.35–2.03)	**<0.001**	–	–
**Talking to friends on phone**				
Yes	1			
No	1.52 (1.22–1.90)	**<0.001**	–	–

α *Mutually adjusted for each other*.

© *Continuous variable*.

*–Not included in multivariate model*.

## Discussion

We conducted a rapid survey to determine the psychological impact of COVID-19 pandemic and its associated factors among the Pakistani general population. This pandemic, apart from the obvious morbidity and mortality, resulted in psychological distress and adverse mental consequences to the population, who are in a constant state of lockdown and quarantine for the last few months (18). The lockdown and curfew measures in Pakistan have affected the lives of common people and as a result triggered psychological problems. Therefore, the estimation of this psychological impact among the general population is crucial in guiding policies and interventions to maintain their psychological well-being. We employed a web-based survey to access the participants throughout Pakistan; it was the first web survey of its kind to assess the psychological impact of the COVID-19 pandemic and its associated factors among the general population of Pakistan.

The finding of our study showed that around 59% of the participants depended on their salary for financial support during the lockdown period. This indicates that 41% of the population depends on sources which can be affected by lockdown. This is important to document that Pakistan is a lower middle income country with limited resources and the major proportion of the population generally struggles to make their ends meet. In this crisis time, when the whole country is shut down, the people living below the poverty line are at high risk of getting affected as they mostly rely on daily wage informal work. There is no effective safety net for the vulnerable population, and therefore, there is an immediate need for the federal and provincial governments to launch the mechanism and programs for the survival of our disadvantage population. On 2nd May 2020, the federal government has launched the Ehsaas program, which promised to provide financial assistance to the unemployed population (19).

Regarding coping strategies adopted by our participants, higher responses were reported for spending quality time with family at home (83%), eating healthy food (79%), taking adequate sleep (77%), and talking to family & friends on the phone (77%). Around two-thirds of the participants reported seeking spiritual support as a coping strategy and 42% of the respondents contributed in social welfare activities. A study from Karachi, Pakistan, reported that religious coping is a common behavior in patients presenting with symptoms of anxiety and depression (20). One recent study in Italy found that people increased the usage of digital media near bedtime during this COVID-19 lockdown (21). As the uncertainty is high regarding the period of this pandemic, the coping strategies during these depressing times are of utmost importance (22). Our population in general is utilizing this lockdown time with useful activities. However, we think that as the closure of work and other activities would continue, the vulnerable population specifically would be requiring psychological support.

In our study, two-thirds of the respondents were hopeful that the COVID-19 pandemic would be successfully controlled in the world, while three-fourths believed that Pakistan would also be able to control this pandemic through mitigation and prevention efforts. A higher proportion was reported in a recent Chinese study, where 90.8% believed that COVID-19 will finally be successfully controlled and 97.1% had confidence that China can win the battle against the virus (23). In our study, more than two-thirds of the respondents (72%) thought that stopping oneself from watching news would help in decreasing the fear related to COVID-19 during this lockdown. It was alarming that a high majority (91%) also believed that unauthentic information through social media is increasing panic about COVID-19. Balkhi et al., in a recent study in Karachi, also reported that a high proportion (84%) believed that fake news surfacing through social media regarding COVID-19 is causing panic (9). As there is an overflow of unconfirmed information related to COVID-19 on social media and there is a tendency of forwarding these types of posts or videos to the social media circle (24), there is a need to strengthen official channels of communication to educate the masses. People should rely on only official resources from the official account of the Ministry of National Health Services Regulations & Coordination, Pakistan, that would help in decreasing the fear and panic related to the pandemic. One third of the participants also thought that social distancing would become the new norm and it would be difficult to meet people in the future like before. This links to the fear and anxiety related to the lockdown and curfew environment, as the pandemic is continuously rising in all parts of the world.

Regarding concerns and worriedness, more than two-thirds of the respondents (72%) were afraid of getting the disease despite preventive measures. This proportion is higher than that reported among the Chinese population (54%) (25). Another recent study from Karachi, Pakistan, reported that around 42% fear for the safety of their health even when they are at home (9). Our study also reported that around 63% of our participants were worried that they would not be able to survive if contracting COVID-19, which is more than in the China study (30%) (25). Around 90% thought that they would transmit the infection to their family members, if they got the infection. This is supplemented by Balkhi et al. (9), who reported that 94% of the respondents fear for the health of their family members in Karachi. Contrastingly, a lower proportion at 75% was reported in a Chinese population related to family members' susceptibility (25). The worriedness and susceptibility about contracting and transmitting infection to oneself and family members was higher in our population. A possible explanation could be that there was no complete compliance by the population to the mitigation measures taken by government, which resulted in fear related to spread of COVID-19.

The prevalence of poor well-being in our study was 41.2%. This finding was similar to one of the large Chinese surveys, which reported 35% of the respondents' experienced psychological distress during this pandemic (7). On the contrary, a recent online survey from Malaysia reported 72.1% moderate to severe anxiety (26), while a study from Egypt found 82% mild to moderate anxiety symptoms during this pandemic (27). The difference observed among different countries regarding psychological well-being could be explained by different healthcare infrastructures, the variability in responsiveness of the health system, and the prevention and control measures taken against the pandemic. The use of different tools to assess psychological well-being during this pandemic also makes it difficult to compare the findings. Regarding risk factors, we found that being a female, being unemployed, residing in Sindh & ICT, fear of COVID-19, and having any chronic illness were significant predictors of poor well-being in our population. Studies conducted in China, Iraq, and Spain reported that female gender was found to be an important predictor of depression, anxiety, and stress as compared to males during the COVID-19 pandemic (7, 28, 29). Another systematic review also reported that female gender was found to be more related to psychiatric disorders (30). Wang et al. also reported that male gender was significantly associated with lower scores in the Impact of Event Scale-Revised (IES-R) as compared to females. Similarly, history of chronic illness was significantly associated with higher IES-R (25, 31), which is also significantly associated with poor well-being in our study. The possible explanation of these findings would be that females as primary caregivers in the households were overburdened with routine household work. In addition, they were also taking care of the responsibilities related to children and male members of the family, who are stuck at home due to lockdown. This made them more vulnerable to poor well-being. Unemployment is also considered as an important factor related to poor well-being, and presence of chronic illness makes the individual vulnerable for psychological issues. People residing in the Sindh & ICT regions were more likely to have poor well-being as compared to the Punjab region. This could be due to differences in socio-political situations and extent of lockdown compliance. However, this needs further exploration. Fear of having COVID-19 and believing that it can be passed to other family members were also significantly associated with poor well-being. Recent studies conducted among the different population groups have also reported that anxiety and fears related to COVID-19 had a significant association with psychological distress and mental health issues (32–35).

We also found that coping strategies; exercise during lockdown; spending time with the family; taking healthy food; having a good sleep; participating in social welfare work; and spending time on their hobbies were significantly associated with better mental well-being. There were few studies conducted on a sample of university students in Poland and China (36, 37), which have reported that certain coping strategies had a positive effect on mental well-being during COVID-19.

We used a standardized and validated Urdu version of the WHO well-being scale for assessing the well-being of the population. As our questionnaire was in both languages (English & Urdu), we were able to include those people who were not comfortable in responding in the English language. We also did factor analysis to extract domains of attitude items, which enabled the use of a standardized tool. Our respondents were from all provinces, and we took the data from all the major cities of Pakistan. Also, there was a fair distribution of respondents from every region of the country. However, there are some limitations that need consideration. The online web survey was able to capture mostly the urban population of Pakistan, so we cannot generalize our findings to the rural population of Pakistan. As it was an online survey, we used all our possible contacts to invite the population through means of phone calls, messages, and one-to-one interactions. There was an oversampling of a particular characteristic (e.g., younger age group, postgraduate, and participants from KPK), leading to selection bias. However, it may serve as an important resource of psychological assessment for the educated and younger population living in Pakistan. Also, we were not able to include those segments of the population who were non-users of social media applications and those who were illiterate. Nevertheless, this is the first study in Pakistan that focused on the impact of the COVID-19 pandemic on psychological well-being of the Pakistani general population from respondents across all regions in the country. Lastly, with the help of our findings, policymakers can develop psychological interventions that can minimize the psychosocial impact of COVID-19 and also help the most vulnerable groups that are at a higher risk of experiencing poor well-being as a result of this pandemic. These findings may also be applicable to other countries with similar socioeconomic profiles as COVID-19 is affecting all the countries and measures of social distancing are more or less similar globally.

## Conclusion

In summary, prevalence of poor well-being due to the COVID-19 pandemic was 41.2% in our population. We reported the initial data pertaining to the psychological impact of the COVID-19 pandemic in the Pakistani population. The predictors of poor well-being were being a female, being unemployed, being a resident of Sindh & ICT, fear of COVID-19, having any chronic illness, and absence of coping strategies. This calls for the development of psychological support and interventions by the policymakers, for the general population as well as for the high-risk groups.

## Data Availability Statement

The raw data supporting the conclusions of this article will be made available by the authors, without undue reservation.

## Ethics Statement

The studies involving human participants were reviewed and approved by Ethics Review Committee of Women Medical College, Abbottabad (20204-2 CMD-ERC-20). The patients/participants provided their written informed consent to participate in this study.

## Author Contributions

AK, FL, and UR conceptualized the study idea, developed methodology, and supervised all the processes. FL and UR performed the data curation. AK and UR performed the data analysis. ZA, FL, AK, UR, SAb, SAr, SI, and MK wrote and reviewed the draft. All authors read and approved the final manuscript.

## Conflict of Interest

The authors declare that the research was conducted in the absence of any commercial or financial relationships that could be construed as a potential conflict of interest.

## References

[1] Li Q Guan X Wu P Wang X Zhou L Tong Y. Early transmission dynamics in Wuhan, China, of novel coronavirus–infected pneumonia. N Engl J Med. (2020) 382:1199–207. 10.1056/NEJMoa2001316 31995857 PMC7121484

[2] Huang C Wang Y Li X Ren L Zhao J Hu Y. Clinical features of patients infected with 2019 novel coronavirus in Wuhan, China. Lancet. (2020) 395:497–506. 10.1016/S0140-6736(20)30183-5 31986264 PMC7159299

[3] World Health Organization WHO Director-General's Opening Remarks at the Media Briefing on COVID-19 - 11 March 2020. (2020). Available online at: https://www.who.int/dg/speeches/detail/who-director-general-s-opening-remarks-at-the-media-briefing-on-covid-19-11-march-2020 (accessed May 15, 2020).

[4] Banerjee D. The COVID-19 outbreak: crucial role the psychiatrists can play. Asian J Psychiatry. (2020) 50:102014. 10.1016/j.ajp.2020.102014 32240958 PMC7270773

[5] Herrman H Saxena S Moodie R Promoting Mental Health: Concepts, Emerging Evidence, Practice: A Report of the World Health Organization, Department of Mental Health and Substance Abuse in Collaboration With the Victorian Health Promotion Foundation and the University of Melbourne. Geneva, Awitzerland: World Health Organization (2005). 10.1037/e538802013-009

[6] Mukhtar S. Mental health and psychosocial aspects of coronavirus outbreak in Pakistan: psychological intervention for public mental health crisis. Asian J Psychiatry. (2020) 51:102069. 10.1016/j.ajp.2020.102069 32344331 PMC7161472

[7] Qiu J Shen B Zhao M Wang Z Xie B Xu Y. A nationwide survey of psychological distress among Chinese people in the COVID-19 epidemic: implications and policy recommendations. Gen Psychiatry. (2020) 33:e100213. 10.1136/gpsych-2020-100213 32215365 PMC7061893

[8] Meng H Xu Y Dai J Zhang Y Liu B Yang H Analyze the psychological impact of COVID-19 among the elderly population in China and make corresponding suggestions. Psychiatry Res. (2020) 289:112983 10.1016/j.psychres.2020.112983 PMC7151427 32388175

[9] Balkhi F Nasir A Zehra A Riaz R. Psychological and behavioral response to the coronavirus (COVID-19) pandemic. Cureus. (2020) 12:e7923. 10.7759/cureus.7923 32499970 PMC7265762

[10] National Institute of Population Studies, NP ICF Pakistan Demographic and Health Survey 2017-18. Islamabad, Pakistan: NIPS Pakistan and ICF (2019).

[11] Dean A Sullivan K Soe M OpenEpi: Open Source Epidemiologic Statistics for Public Health, Version 2013. Available online at: www.OpenEpi.com (accessed April 15, 2020).

[12] Bech P Gudex C Johansen KS. The WHO (Ten) well-being index: validation in diabetes. Psychotherapy Psychosomatics. (1996) 65:183–90. 10.1159/000289073 8843498

[13] Warr P Banks M Ullah P. The experience of unemployment among black and white urban teenagers. Br J Psychol. (1985) 76:75–87. 10.1111/j.2044-8295.1985.tb01932.x 3978357

[14] Krieger T Zimmermann J Huffziger S Ubl B Diener C Kuehner C. Measuring depression with a well-being index: further evidence for the validity of the WHO Well-Being Index (WHO-5) as a measure of the severity of depression. J Affective Disord. (2014) 156:240–4. 10.1016/j.jad.2013.12.015 24412323

[15] Schneider CB Pilhatsch M Rifati M Jost WH Wodarz F Ebersbach G. Utility of the WHO-five well-being index as a screening tool for depression in Parkinson's disease. Mov Disord. (2010) 25:777–83. 10.1002/mds.22985 20108365

[16] World Health Organization WHO-5 Questionnaires: Urdu Version. Available online at: https://www.psykiatri-regionh.dk/who-5/Documents/WHO5_Urdu.pdf (accessed April 14, 2020).

[17] Topp CW Østergaard SD Søndergaard S Bech P. The WHO-5 well-being index: a systematic review of the literature. Psychotherapy Psychosomatics. (2015) 84:167–76. 10.1159/000376585 25831962

[18] Brooks SK Webster RK Smith LE Woodland L Wessely S Greenberg N. The psychological impact of quarantine and how to reduce it: rapid review of the evidence. Lancet. (2020) 395:912–20. 10.1016/S0140-6736(20)30460-8 32112714 PMC7158942

[19] Government of Pakistan Ehsaas Program, Division of Poverty Alleviation and Social Safety. Available online at: https://www.pass.gov.pk/ (accessed May 19, 2020).

[20] Kasi PM Naqvi HA Afghan AK Khawar T Khan FH Khan UZ. Coping styles in patients with anxiety and depression. ISRN Psychiatry. (2012) 2012:128672. 10.5402/2012/128672 23738194 PMC3658553

[21] Cellini N Canale N Mioni G Costa S. Changes in sleep pattern, sense of time, and digital media use during COVID-19 lockdown in Italy. J Sleep Res. (2020) 10.31234/osf.io/284mr 32410272 PMC7235482

[22] Wood W Rünger D. Psychology of habit. Ann Rev Psychol. (2016) 67:289–314. 10.1146/annurev-psych-122414-033417 26361052

[23] Zhong B-L Luo W Li H-M Zhang Q-Q Liu X-G Li W-T. Knowledge, attitudes, and practices towards COVID-19 among Chinese residents during the rapid rise period of the COVID-19 outbreak: a quick online cross-sectional survey. Int J Biol Sci. (2020) 16:1745. 10.7150/ijbs.45221 32226294 PMC7098034

[24] BSD RP Wilder-Smith A. The pandemic of social media panic travels faster than the COVID-19 outbreak. J Travel Med. (2020) 27:taaa031. 10.1093/jtm/taaa031 32125413 PMC7107516

[25] Wang C Pan R Wan X Tan Y Xu L Ho CS. Immediate psychological responses and associated factors during the initial stage of the 2019 coronavirus disease (COVID-19) epidemic among the general population in China. Int J Environ Res Public Health. (2020) 17:1729. 10.3390/ijerph17051729 32155789 PMC7084952

[26] Wong LP Alias H. Temporal changes in psychobehavioural responses during the early phase of the COVID-19 pandemic in Malaysia. J Behav Med. (2020) 51:92–93. 10.1007/s10865-020-00172-z 32757088 PMC7405711

[27] Magdy DM Metwally A Magdy O Assessment of Community Psycho-behavioral Responses during the outbreak of novel coronavirus (2019-nCoV): a cross sectional study. Res Square [preprint]. (2020). 10.21203/rs.3.rs-25146/v1 PMC8755960 35071666

[28] Othman N Depression, anxiety, and stress in the time of COVID-19 pandemic In Kurdistan Region, Iraq. Kurdistan J Appl Res. (2020) 5:37–44. 10.24017/covid.5

[29] González-Sanguino C Ausín B ÁngelCastellanos M Saiz J López-Gómez A Ugidos C. Mental Health Consequences during the Initial Stage of the 2020 Coronavirus Pandemic (COVID-19) in Spain. Brain Behav Immun. (2020) 87:172–6. 10.1016/j.bbi.2020.05.040 32405150 PMC7219372

[30] Pappa S Ntella V Giannakas T Giannakoulis VG Papoutsi E Katsaounou P. Prevalence of depression, anxiety, and insomnia among healthcare workers during the COVID-19 pandemic: a systematic review and meta-analysis. Brain Behav Immun. (2020) 88:901–7. 10.2139/ssrn.3594632 32437915 PMC7206431

[31] Ozamiz-Etxebarria N Dosil-Santamaria M Picaza-Gorrochategui M Idoiaga-Mondragon N. Stress, anxiety, and depression levels in the initial stage of the COVID-19 outbreak in a population sample in the northern Spain. Cadernos de Saúde Pública. (2020) 36:e00054020. 10.1590/0102-311x00054020 32374806

[32] Zaki WN Sidiq M Qasim M Aranas B Hakamy A Ruwais N Stress and psychological consequences of COVID-19 on health-care workers. J Nat Sci Med. (2020) 3:299–307. 10.4103/JNSM.JNSM_86_20

[33] Kecojevic A Basch CH Sullivan M Davi NK. The impact of the COVID-19 epidemic on mental health of undergraduate students in New Jersey, cross-sectional study. PLOS ONE. (2020) 15:e0239696. 10.1371/journal.pone.0239696 32997683 PMC7526896

[34] Khan AH Sultana MS Hossain S Hasan MT Ahmed HU Sikder MT. The impact of COVID-19 pandemic on mental health & wellbeing among home-quarantined Bangladeshi students: a cross-sectional pilot study. J Affect Disord. (2020) 277:121–8. 10.1016/j.jad.2020.07.135 32818775 PMC7410816

[35] Alkhamees AA Alrashed SA Alzunaydi AA Almohimeed AS Aljohani MS. The psychological impact of COVID-19 pandemic on the general population of Saudi Arabia. Compr Psychiatry. (2020) 102:152192. 10.1016/j.comppsych.2020.152192 32688022 PMC7354380

[36] Rogowska AM Kuśnierz C Bokszczanin A. Examining anxiety, life satisfaction, general health, stress and coping styles during COVID-19 pandemic in polish sample of University Students. Psychol Res Behav Manage. (2020) 13:797–811. 10.2147/PRBM.S266511 33061695 PMC7532061

[37] Nurunnabi M Hossain SFAH Chinna K Sundarasen S Khoshaim HB Kamaludin K. Coping strategies of students for anxiety during the COVID-19 pandemic in China: a cross-sectional study. F1000Res. (2020) 9:1115. 10.12688/f1000research.25557.1 33274049 PMC7682494

