# Supplementary material for: Impact of Coronavirus Disease (COVID-19) Pandemic on Psychological Well-Being of the Pakistani General Population
Source: Front Psychiatry. 2021 Jan 12;11:564364. doi: 10.3389/fpsyt.2020.564364 (PMC7835389; doi:10.3389/fpsyt.2020.564364)
Supplement: Supplementary file 1 [file Table_1.DOCX]

| **Supplementary Table 1: Wellbeing of Pakistani general population during COVID-19 pandemic (n=1756)** | |
| --- | --- |
| **Variable** | **n (%)** |
| **I have felt cheerful and in good spirit (n=1720)**  At no time  Some of the time  Less than half of the time  More than half of the time  Most of the times  All of the time | 106 (6.2)  457 (26.6)  176 (10.2)  306 (17.8)  508 (29.5)  167 (9.7) |
| **I have felt calm and relaxed (n=1725)**  At no time  Some of the time  Less than half of the time  More than half of the time  Most of the times  All of the time | 82 (4.8)  395 (22.9)  169 (9.8)  334 (19.4)  566 (32.8)  179 (10.4) |
| **I have felt active and vigorous (n = 1719)**  At no time  Some of the time  Less than half of the time  More than half of the time  Most of the times  All of the time | 124 (7.2)  447 (26.0)  239 (13.9)  299 (17.4)  457 (26.6)  153 (8.9) |
| **I woke up feeling fresh and rested (n=1728)**  At no time  Some of the time  Less than half of the time  More than half of the time  Most of the times  All of the time | 120 (6.9)  323 (18.7)  181 (10.5)  270 (15.6)  617 (35.7)  217 (12.6) |
| **My life is filled with things which interest me (n=1713)**  At no time  Some of the time  Less than half of the time  More than half of the time  Most of the times  All of the time | 107 (6.2)  408 (23.8)  205 (12.0)  272 (15.9)  521 (30.4)  200 (11.7) |
